# Severe hand contracture treated by external fixation after proximal row carpectomy and awake tenolysis after general anesthesia: A case report

**DOI:** 10.1016/j.cjtee.2021.08.008

**Published:** 2021-08-16

**Authors:** Takuya Tsumura, Taiichi Matsumoto, Mutsumi Matsushita, Katsuma Kishimoto, Hayao Shiode, Hiroshi Murakami

**Affiliations:** Department of Orthopedic Surgery, Kurashiki Central Hospital, 1-1-1 Miwa, Kurashiki, 710-0052, Japan

**Keywords:** General anesthesia, Ilizarov technique, Tendons, Carpectomy

## Abstract

Wide-awake local anesthesia has many advantages. We describe a new method to use wide-awake local anesthesia with more flexibility. A 32-year-old man with a severe right-hand contracture after an iatrogenic tourniquet accident during an anterolateral thigh flap for a partial hand amputation underwent contracture release using external fixation after proximal row carpectomy and subsequent tenolysis. We performed most of the tenolysis procedure under general anesthesia and the final stage with an intraoperative assessment of active finger movement and dissection under local anesthesia. He regained his grip strength 2.5 years post-injury. General anesthesia is useful to treat a surgical site with extensive hard scars, whereas local anesthesia is useful for adjusting tension in an awake patient. The indication for wide-awake surgery is yet to be established; our method of combining general and local anesthesia in the tenolysis procedure illustrates the possibilities in expanding this method.

## Introduction

Severe hand trauma often causes severe hand contracture. Performing tenolysis after open contracture release is difficult because of extensive hard scars, in which damage to the reconstructed vessels and nerves is hard to prevent. To minimize scar formation and tendon adhesions, we performed contracture release using external fixation after proximal row carpectomy (PRC) before tenolysis. Regarding the tenolysis procedure, one of the advantages of performing wide-awake surgery is that it allows surgeons to assess active finger movement.^1^^,^^2^ For the treatment of severe trauma with extensive hard scars, an increased volume of anesthesia may be necessary, and soft tissue dissection may injure the reconstructed vessels and nerves. Also, awake patients often complain about lower back pain caused by being in the supine position for an extended period of time. To solve these problems, we developed a new method for tenolysis in which most of the procedure is performed under general anesthesia with only the final stage performed with intraoperative assessment of active finger movement and dissection under local anesthesia. Here, we describe a case of severe hand contracture treated by external fixation after PRC and awake tenolysis after general anesthesia.

## Case report

**Fig. 1:**
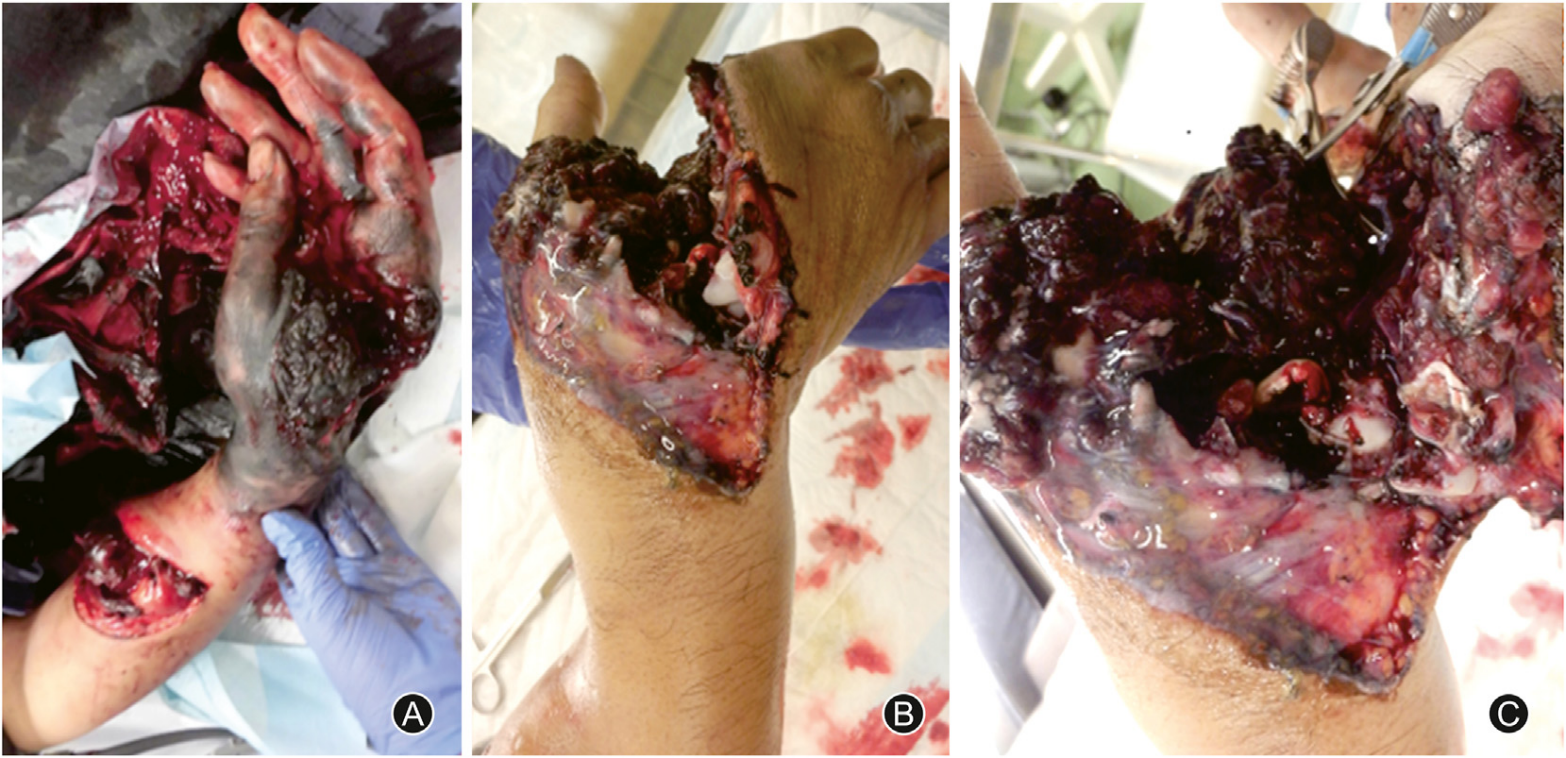
The day of injury. (A) The right hand is contaminated with machine oil. (B) The hand is partially amputated. (C) Thorough debridement is performed to prevent infection.

**Fig. 2:**
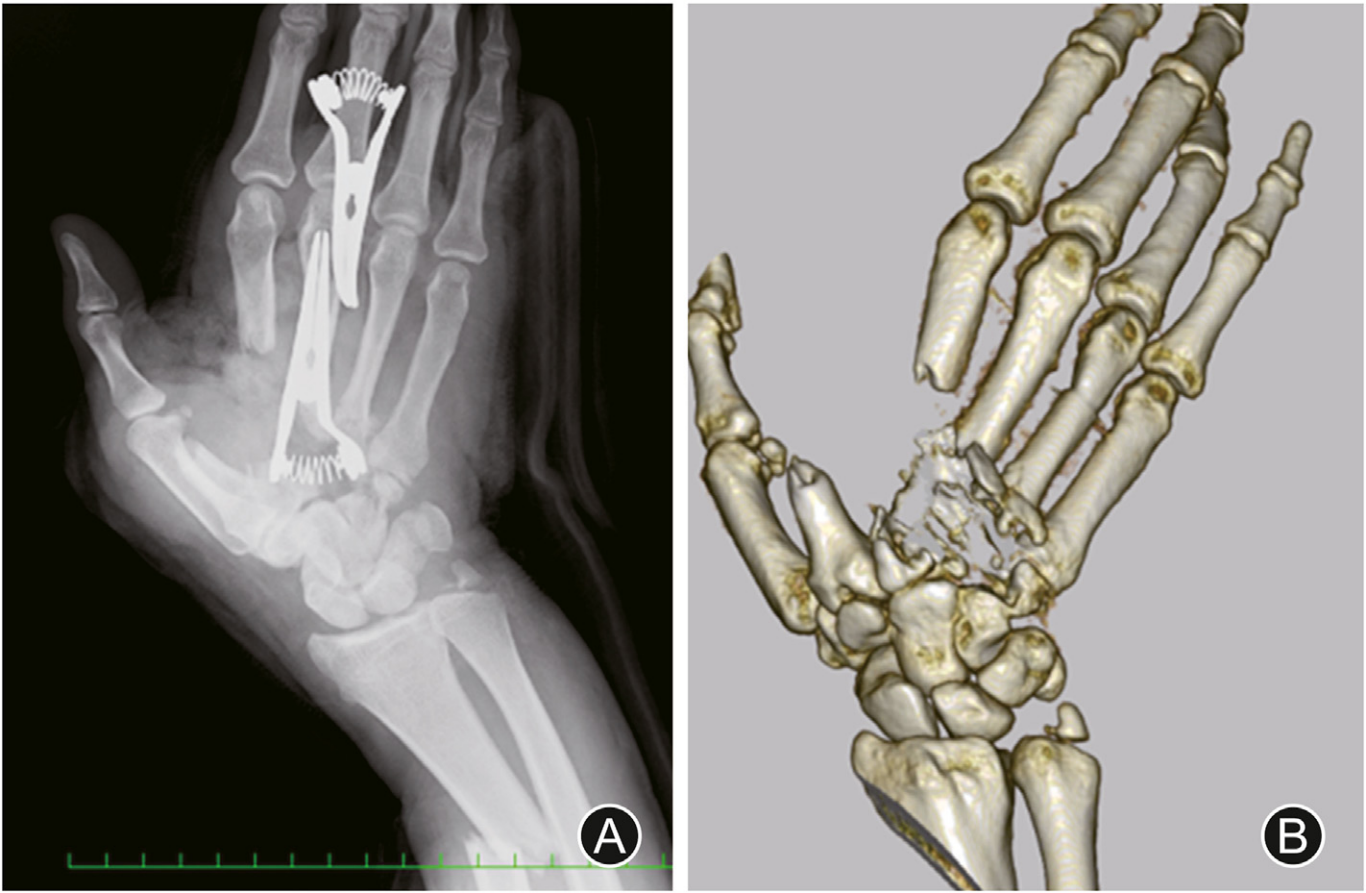
Images of the second, third, and fourth metacarpal bone fractures (A: radiograph, B: CT scan).

**Fig. 3:**
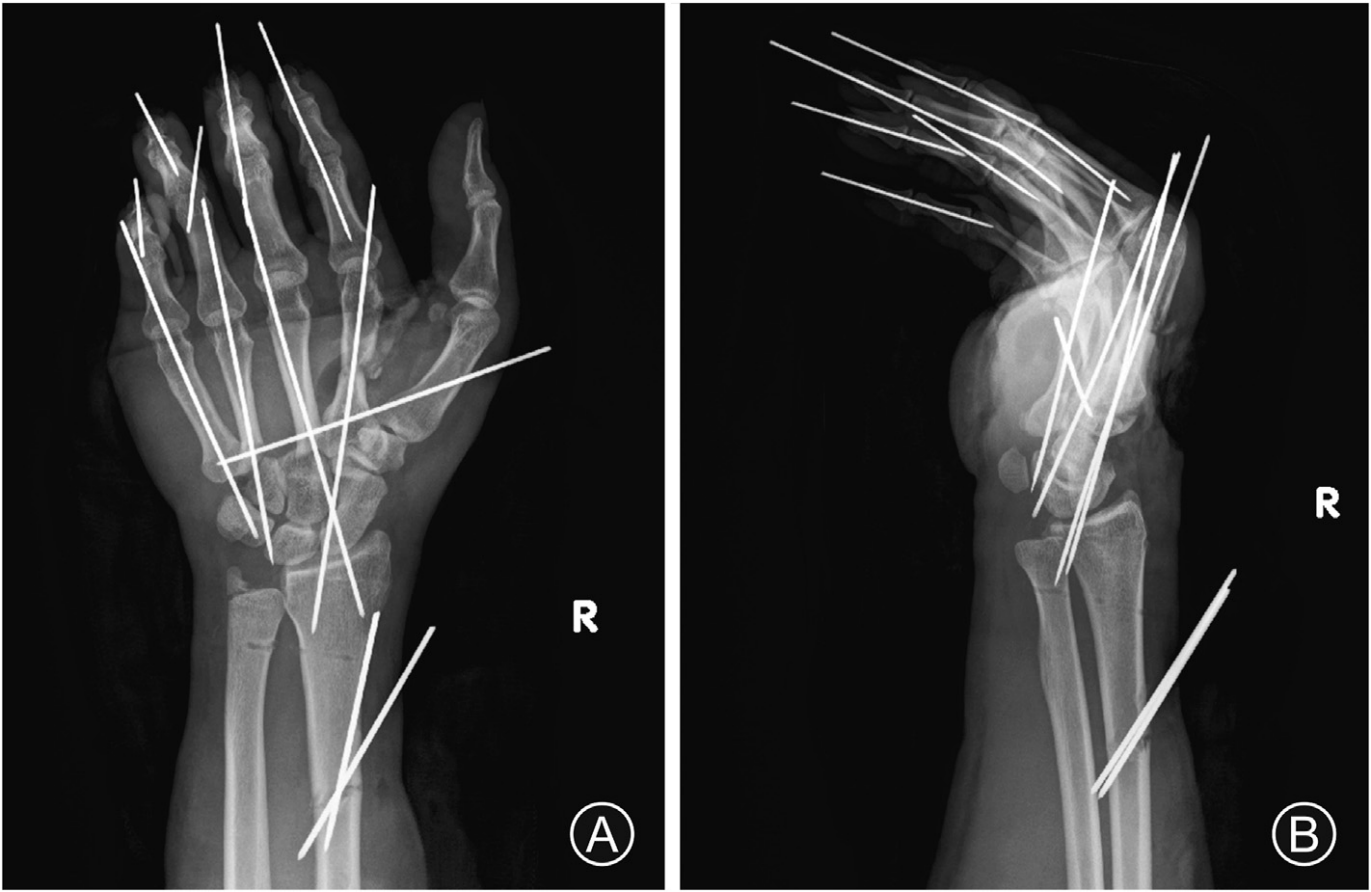
Radiographs showing the metacarpophalangeal joints fixed with block pins and the distal interphalangeal and passive proximal interphalangeal joints temporarily fixed with a K-wire (A: frontal view, B: lateral view).

A 32-year-old man was struck by a milling cutter and sustained a partial right-hand amputation (Fig. 1). Radiography and CT revealed fractures of the second, third, and fourth metacarpal bones (Fig. 2). Traumatic features of this case include a forearm radial artery defect of 10 cm, rupture of the princes pollicis and the first and second common digital arteries, nerve rupture in the thumb, index, and middle fingers, and defects in the recurrent branch of the median nerve and the deep branch of the ulnar nerve. The defects in the recurrent branch of the median nerve and the deep branch of the ulnar nerve were left unrepaired because they were pulled out and severely damaged. The forearm radial, princeps pollicis, and common digital arteries were repaired with small saphenous vein grafts, and the digital nerves with saphenous nerve grafts. The extensor pollicis longus and flexor carpi ulnaris remained, but the flexor carpi radialis, flexor pollicis longus, and all the flexor digitorum superficialis, flexor digitorum profundus, extensor digitorum communis, and extensor indicis muscles had ruptured. The extensor and flexor tendons were completely contaminated with machine oil; therefore, thorough debridement was performed at the tendon edge. We resected the flexor digitorum superficialis, lumbricals, and interossei muscles because of irreparable contamination, and sutured the flexor digitorum profundus, flexor pollicis longus, and extensor digitorum communis using a 6-strand suture technique. Tendon shortening increased tension in the extensor and flexor tendons. Although the fingers were passively extended, a spring-like resistance occurred when they were extended. The flexor tendons of the fingers became so rigid after thorough debridement that even passive extension was difficult, resulting in a clenched fist and flexion contracture. To treat this deformity, we inserted block pins into the metacarpal bones, fixed the metacarpophalangeal (MP) joints in a flexion position, and temporarily fixed the distal interphalangeal and passive proximal interphalangeal (PIP) joints with a K-wire in an extension position to keep them in a functional position (Fig. 3).

Ten days post-injury, we performed an anterolateral thigh flap to cover the skin defects. Although we did not use a tourniquet, we wrapped one around the upper arm as a precaution. After flap elevation, we tried to anastomose the descending branch with the ulnar artery, but could not palpate the ulnar artery pulsation, because the tourniquet was accidently inflated. After the flap surgery, we analyzed the surgical video, but could not determine why and for how long the tourniquet had been inflated. The postsurgical course was unfavorable; the patient could not move his right upper limb, and his forearm was significantly swollen. Three days after the flap surgery, the biceps and triceps strength recovered to manual muscle testing (MMT) grade 2, and the patient was able to move his fingers slightly. We removed the temporary K-wire fixation. Seven days after the flap surgery, the biceps and triceps strength recovered to MMT grade 4, and his finger strength to MMT grade 1.

Five months post-injury, hand contracture resembling intrinsic plus hand persisted, and the active range of motion (ROM) was limited. The biceps and triceps strength recovered to MMT grade 5, and the finger strength to MMT grade 3. The intrinsic tightness test was positive. When the MP joints were extended, PIP flexion slightly improved by about 5°. Even when the fingers were passively flexed, only flexion of about 20° was obtained, which was far from adequate. The wrist and fingers were in severe contracture, and the finger strength was MMT grade 3. Tendon transfer was not performed because of the requirement for supple joints and adequate muscle strength. We performed PRC to reduce wrist contracture and tightness in flexion and extension. After PRC, the ROM of the PIP joints improved, but that of the wrist remained unchanged.

**Fig. 4:**
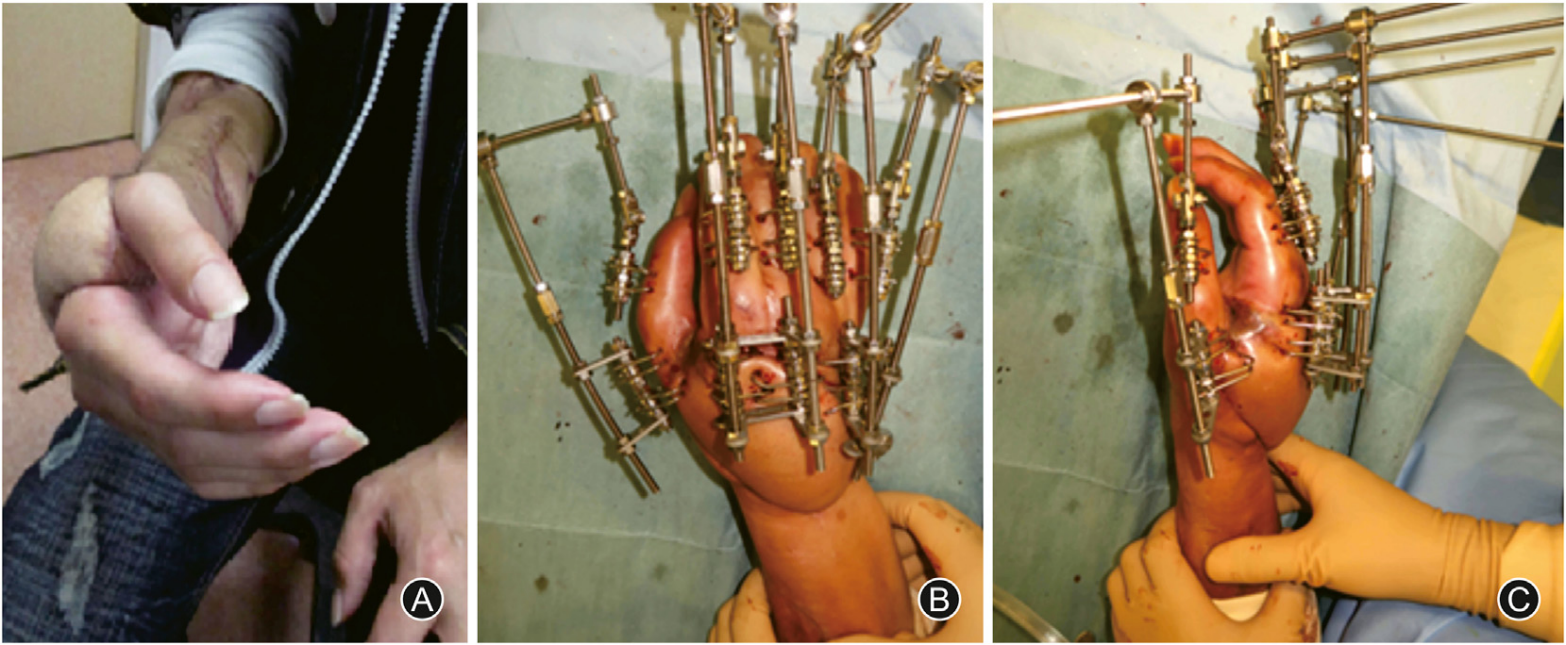
(A) The patient's right-hand contracture resembling an intrinsic plus hand. Distraction arthroplasty is performed for metacarpophalangeal joint and thumb-index adduction contracture release using an Ilizarov mini fixator (B: anteroposterior view, C: lateral view).

**Fig. 5:**
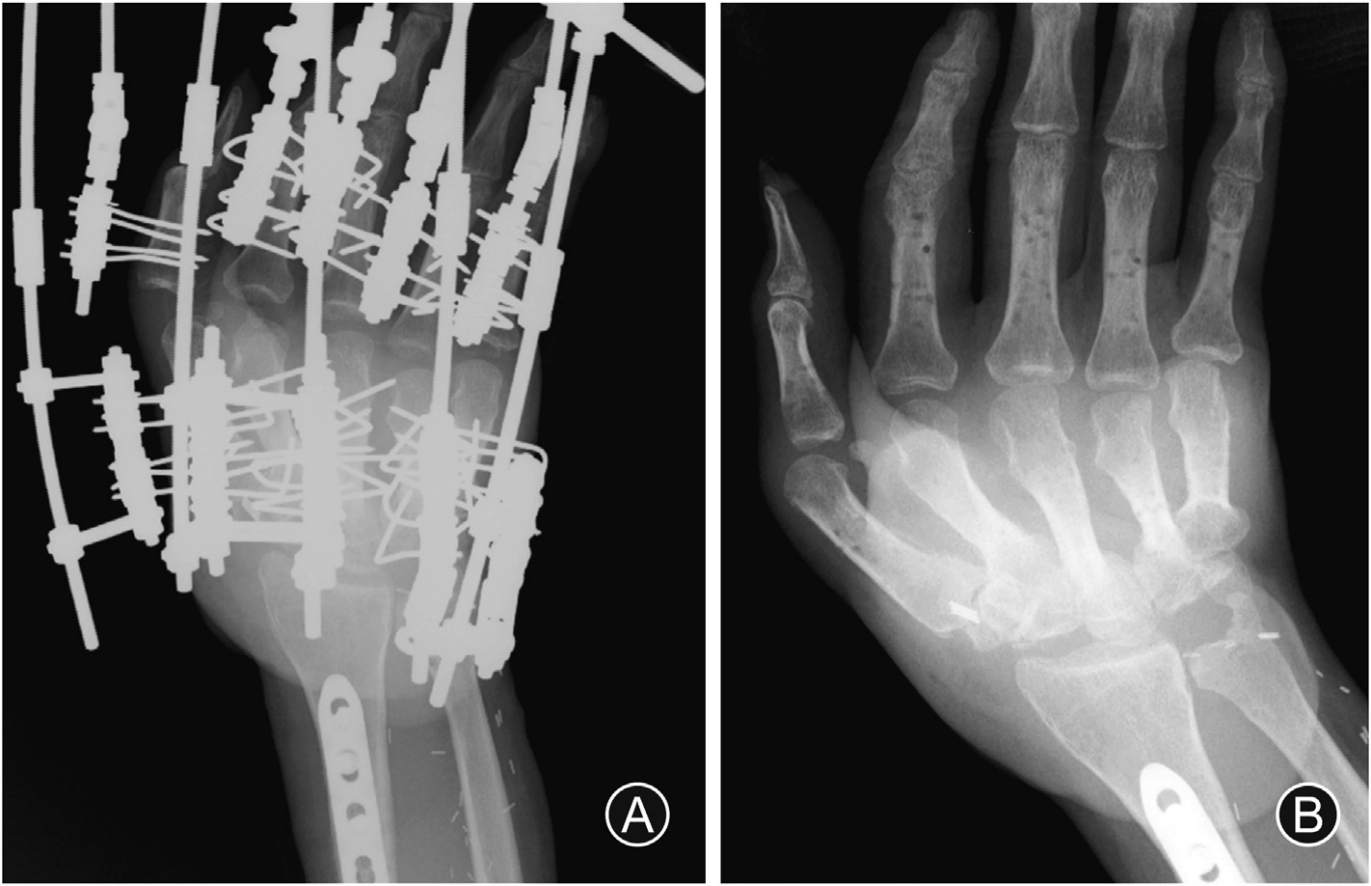
Radiograph showing contracture release (A: with an Ilizarov mini fixator, B: after external fixator removal).

Seven months post-injury, we performed distraction arthroplasty for the MP joint and thumb-index adduction contracture release using an Ilizarov mini fixator (ARATA Co., Ltd., Tokyo, Japan) (Fig. 4, Fig. 5). After distraction arthroplasty, passive ROM improved; however, active ROM was so small that the patient could not grasp anything (video file available on YouTube at https://youtu.be/u7i3dR7841A).

One year post-injury, the patient's fingers recovered to MMT grade 5, but were far from adequate. His quick disabilities of the arm, shoulder and hand (DASH) score was 59.09 in the disability and symptoms category. His grip strength was zero because he was unable to grasp anything. Although tenolysis was necessary, performing it in an awake state was difficult because of the extensive hard scars formed after the vessel and nerve repair. We therefore decided to perform most of the tenolysis procedures under general anesthesia and the final dissection under local anesthesia. First, we placed the patient in the supine position and anesthetized him with desflurane inhalation. Then, we injected 0.5% lidocaine with epinephrine and 0.75% ropivacaine subcutaneously with a 22-G needle to reduce bleeding. While scrubbing and draping the patient, the anesthetic agents penetrated the surgical site adequately. To reduce postoperative swelling, we used a tourniquet as minimally as possible. We made an incision from slightly distal to the MP joints to the proximal wrist joint. We performed most of the tenolysis procedures under general anesthesia. Before entering the final stage, we asked the anesthesiologist to prepare for discontinuation of inhalation. Upon completion of tenolysis, we asked the anesthesiologist to awaken the patient. Four min after extubation, he emerged from anesthesia, and we asked him to perform full-finger flexion. Because the movement of the index finger was inferior to those of the other fingers, we performed additional tenolysis as wide-awake surgery, without any additional anesthetic injection (video file available on YouTube at https://youtu.be/ojN8hQKYN_8). The surgical time was 4 h and 23 min.

After tenolysis, the patient was able to perform active finger movement. The total active motion of the thumb, index, middle, ring and little fingers improved from 4%, 12%, 13%, 17% and 17% preoperatively to 14%, 48%, 54%, 45% and 56% postoperatively. Two and a half years post-injury, his grip strength recovered to 14 kg, and his quick DASH score was 20 in the disability and symptoms category, and zero in the work category (video file available on YouTube at https://youtu.be/N1irw9EantQ).

## Discussion

The main cause of intrinsic plus hand is intrinsic muscle contracture. In this case, the palmar interosseous and lumbrical muscles were resected in the first debridement; however, the dorsal interosseous muscles were retained. This was not a simple intrinsic plus contracture caused by only intrinsic muscle contracture. The intrinsic plus like contracture of the patient's hand might have been due to that of the dorsal intrinsic muscles and local compartment syndrome caused by the tourniquet accident or transient MP joint block pins and tightness of the extensor and flexor tendons.

Advancing interosseous muscles, resecting oblique fibers of the extensor expansion hood (distal intrinsic transfer), and resecting interosseous and lumbrical muscles in the musculotendinous portion (proximal intrinsic transfer) are treatment options for intrinsic plus contracture.^3^, ^4^, ^5^ Because the PIP joints remained fixed during the Bunnell-Littler test regardless of whether the MP joints were fully flexed or extended, the intrinsic plus contracture was considered to be caused by multiple factors, including PIP joint contracture, intrinsic contracture, extensor and flexor tendon shortening, and adhesions. In this case, the proximal intrinsic release was not useful because the volar interosseous and lumbrical muscles were almost completely resected during the first surgery through debridement, and distal intrinsic release was considered useless because of MP joint flexion contracture. Open surgery such as open contracture release and tendon transfer require extensive dissection and was considered not suitable due to the possibility of inducing further contracture. Because the patient had almost no passive wrist joint and finger ROM, performing tendon transfer alone seemed to be of no use.

Hamada et al.^6^ reported successful treatment of severe contracture of intrinsic plus hand with an Ilizarov mini fixator after reducing the tension on the extrinsic muscle by PRC. Similarly, we performed PRC first to reduce the shortening of the extensor and flexor tendon and wrist contracture. After the PIP joint contracture was released, the MP joint contracture was released by external fixation. Resolving the shortening of the tendon with external fixation alone seemed to be difficult. Also, treatment of the PIP and wrist joint contracture with external fixation seemed to be difficult because an external fixator was necessary for the PIP and wrist joints, respectively, which required more time and cost.

Usually, tenolysis performed under upper arm block anesthesia or general anesthesia. Adequacy of complete tendon release was confirmed by pulling the proximal part of the tendon and obtaining a preoperative passive ROM. However, it can be different from the real excursion and gliding of the tendon *in vivo*. This is because it is not possible to manually pull a tendon with the same force as a living organism. Therefore, it is necessary to confirm the active ROM in wide–awake surgery. If the muscle power is inadequate or the tendon is elongated, another tendon reconstruction surgery is necessary. A longer than usual surgical time associated with the presence of extensive hard scars discouraged us from performing wide-awake surgery. We decided to perform most of the tenolysis procedures under general anesthesia and the final stage under local anesthesia so that we would be able to assess active finger movement. We locally injected analgesics immediately after intubation, which contained a long-acting analgesic, namely, ropivacaine. Using ropivacaine seemed to bring about the desired effect because the amount of anesthetic or narcotics required during surgery was reduced, which could achieve a rapid wide awakening. Because there was a concern about delirium as a side effect, we explained the intraoperative awakening to the patient before the surgery. A restraining band was used during extubation, and the affected limb was held down by the operator's hand to avoid contamination of the surgical field. The urethral catheter was removed before extubation. We used desflurane as a general anesthetic because of its rapid emergence.^7^, ^8^, ^9^

Until recently, wide-awake surgery has been used mainly in simple surgeries such as tendon tenolysis, tendon transfer, metacarpal fracture, and carpometacarpal joint arthroplasty.^10^ Our new method offers some advantages over simple wide-awake surgery. It can be used in complicated tendon surgeries in which a longer than usual surgical duration is expected and a tourniquet can be used. In addition to tenolysis, tendon transfer can also be performed using this method which allows surgeons to assess tendon tension intraoperatively. Furthermore, complex tendon surgeries such as triple tendon transfer for radial nerve reconstruction and tendon surgery for ulnar nerve reconstruction can be performed using this new method.

The indication for wide-awake surgery is yet to be established; our method of combining general and local anesthesia in tenolysis illustrates the possibility for expanding of this method.

## Funding

This research did not any specific grant from funding agencies in the public, commercial, or not-for-profit sectors.

## Ethical statement

Informed consent was obtained from the patient for publication of this case and the associated images. All procedures were in accordance with the ethical standards of the responsible committee on human experimentation (institutional and national) and with the Helsinki Declaration.

## Declaration of competing interest

None.
